# Annotations

**Published:** 1908-05-09

**Authors:** 


### Annotations.

### The Tsetse-Fly Diseases.

Since the adoption by Germany of her Colonial policy, she has been confronted by problems in tropical hygiene and medicine whose investigation was formerly almost the monopoly of our own country. Since Professor Robert Koch's work on rinderpest a decade ago, he has rightly been regarded as one of the highest living authorities on the bacteriology of the African diseases. His latest pronouncements on the human and animal diseases carried by various species of tsetse fly have aroused considerable opposition, however, not only in this country, but in Germany also. From the reports of a recent meeting in Berlin it seems that Professor Koch has proposed the systematic extermination of big game in the German African colonies in order to eliminate the tsetse fly, and that the proposal has brought a hornet's nest of outraged naturalists, dissenting protozoologists, and agricultural economists about his ears. The worth of this suggestion must, of course, be determined by the question of its probable efficacy alone, but there are certain important considerations to be borne in mind. For it is held by competent authorities that not only does the carrier of human trypanosomiasis thrive on the blood both of wild and domestic animals, but also that the carrier of bovine and equine trypanosomiasis thrives on a diet of man. It is therefore of no great value either to man or to his ox, ass, pig, and other stock, to destroy the wild game only. It is highly desirable that the mass of assertion and counter-assertion on these fly-borne plagues should be penetrated by scientific investigation; and until more reliable information as to the exact life history both of the parasites and their carriers is available, it is futile to advocate prophylactic measures which involve the irrevocable sacrifice of assets important to the heavily-pressed African protectorates.

### Preservatives in Food.

The more we hear of the evils, real and supposed, arising from the addition of large quantities of chemical preservatives to food stuffs, the more important does it seem that there should be a fixed legal limit as to the amount and nature of preservative which may be used. Dr. Collingridge, the medical officer of health for the City of London, drew attention just over a month ago to a consignment of kidneys from America, which contained very large quantities of boracic acid. He now reports upon samples of Danish kidneys which have recently been examined. Out of twelve samples, only two were found to contain preservatives in excessive quantities. It is interesting to note in this connection that there is a Bill now before the Danish Government, seeking power to prohibit the use of preservatives of any kind in such goods, as they are believed by the authorities in that country to be absolutely unnecessary and objectionable. Four samples of cooked tripe from the United States have also been examined, and they, like the American kidneys, showed the presence of boracic acid in great excess. As Dr. Collingridge remarks, there is not likely to be much difference of opinion as to the advisability of preventing the sale of tripe containing 77 grains of boric acid in an average meal of half a pound. It may seem somewhat hard on the American consignors that, in the absence of any legal standard, their goods should run the risk of wholesale destruction on their arrival in England; but 154 grains of boracic acid per pound of meat cannot be excused. They have now received instructions by cable to discontinue sending these food-stuffs to the London markets. But the matter cannot very well end here, and we look forward to an early official pronouncement upon the whole subject. The toxic possibilities of boracic acid may be somewhat ill-defined, and expert opinion may still be divided as to how much of this salt can be taken with impunity; but a minimum could surely be agreed upon as a preliminary working standard.

### Altitude and Health.

Although the subject of mountain sickness has been carefully studied at different times, and reported upon by skilled observers such as Whymper and Paul Bert, the effects of prolonged residence in high localities have not received the same attention from scientists. The researches of De Sassure, Marcet, and Viault do not seem to have been carried by later observers beyond the physiological results of more or less rapid changes of altitude. It is taken for granted by most writers that after a certain length of time a healthy man can adapt himself perfectly to any degree of altitude. Medical practitioners resident in elevated parts of South Africa have, however, lately cast doubts on the ideas which are usually held on this subject. Observations on persons apparently well-acclimatised to an elevation of 6,000 feet above the coast-level frequently reveal a constant increase in the pulse-rate, while the examination of a number of Johannesburg school-children showed a large proportion of cases of cardiac hypertrophy. As a result of these observations, the Transvaal Medical Society has appointed a sub-committee to investigate the clinical and physiological effects of altitude upon persons resident in high localities. Remembering how many and how varied have been the factors alleged by different workers to be essential to the production of mountain sickness, and how little is still known, in spite of repeated experiments, of the physiological effects of rapid ascents, we must not expect too much of this inquiry into the influence upon the body of prolonged exposure to high altitudes. But the sub-committee of the Transvaal Medical Society should be able to determine whether the alleged changes in the circulatory system are really present, and, if so, whether they bear a constant relation to height above the sea-level.

